# Enhancing neural markers of attention in children with ADHD using a digital therapeutic

**DOI:** 10.1371/journal.pone.0261981

**Published:** 2021-12-31

**Authors:** Courtney L. Gallen, Joaquin A. Anguera, Molly R. Gerdes, Alexander J. Simon, Elena Cañadas, Elysa J. Marco

**Affiliations:** 1 Department of Neurology, University of California, California, San Francisco, United States of America; 2 Neuroscape Center, University of California, California, San Francisco, United States of America; 3 Department of Psychiatry, University of California, California, San Francisco, United States of America; 4 Department of Neurodevelopmental Medicine, Cortica Healthcare, San Rafael, CA, United States of America; 5 Akili Interactive Labs, Boston, MA, United States of America; 6 Department of Radiology, University of California, California, San Francisco, United States of America; Osaka University Graduate School of Medicine, JAPAN

## Abstract

Attention deficit hyperactivity disorder (ADHD) is a prevalent neurodevelopmental condition characterized by diminished attentional control. Critically, these difficulties are related to negative consequences in real-life functioning both during development and into adulthood. There is now growing evidence that modulating the underlying neural circuits related to attention can improve behavior and brain function in children with ADHD. We have previously shown that game-based digital therapeutics targeting a key neural marker of attention–midline frontal theta (MFT)–yield positive effects on attentional control in several populations. However, the effects of such digital therapeutics in children with ADHD and no other comorbidities has not been yet examined. To address this gap, we assessed a sample of 25 children with ADHD (8–12 years old) on neural, behavioral, and clinical metrics of attention before and after a 4-week at-home intervention on an iPad targeting MFT circuitry. We found that children showed enhancements on a neural measure of attention (MFT power), as well as on objective behavioral measures of attention and parent reports of clinical ADHD symptoms. Importantly, we observed relationships between the neural and behavioral cognitive improvements, demonstrating that those children who showed the largest intervention-related neural gains were also those that improved the most on the behavioral tasks indexing attention. These findings provide support for using targeted, digital therapeutics to enhance multiple features of attentional control in children with ADHD.

**Study registration:** ClinicalTrials.gov registry (NCT03844269) https://clinicaltrials.gov/ct2/show/NCT03844269.

## Introduction

Attention-deficit hyperactivity disorder (ADHD) is one of the most common neurodevelopmental conditions affecting children, with an estimated 6.1 million youth aged 2–17 years (9.4%) in the United States ever having received this diagnosis [1]. Individuals with ADHD exhibit persistent patterns of diminished attentional control–the capacity to appropriately allocate attention resources and monitor the environment for new sources of information, ultimately enabling optimal processing of task-relevant information [2]. Attentional control is a key component of general cognitive control abilities that are critical for goal-directed behavior [3, 4]. Importantly, deficits in attentional control interfere with cognitive and behavioral development and impact function at home, school, and in the community [5–7].

The current recommended treatments for ADHD include pharmacological and behavioral therapies [8, 9]. Although these treatments show some promise at ameliorating ADHD symptoms, they also have several limitations. For example, pharmacological treatments often induce side effects such as reduced appetite [10] and there are barriers to obtaining non-pharmacological (behavioral) interventions [11, 12]. There is growing evidence, however, that modifying brain function through repeated practice can also improve attention, across age and mental health conditions, as an alternative or adjunct to prescription medication [13].

Video game-based interventions that target the underlying neural mechanisms of attentional control abilities are one example of neuromodulation for ameliorating cognitive dysfunction. Several studies have previously investigated the efficacy of multi-tasking training delivered by a video-game based experience, where participants simultaneously perform a perceptual discrimination task (similar to a go/no-go task) and a sensory motor navigation task. This work has demonstrated that multi-tasking training that targets midline frontal theta (MFT) circuitry can improve behavior and brain function across a range of populations. MFT (4-7Hz) power is a well-described neural signature of attention and broader cognitive control abilities that has been associated with signaling the need for increased attention in a variety of contexts [14, 15]. Using electroencephalography (EEG) [16], we initially showed that training on a custom-designed multi-tasking video game, *NeuroRacer*, remediated age-related neural deficits in MFT and had positive effects on related objective behavioral metrics of attention and other cognitive control abilities in healthy older adults [17]. Next, we extended this work to children with attention difficulties using a digital therapeutic based on *NeuroRacer–*AKL-T01. We showed that AKL-T01 improved MFT and behavioral measures of attentional control in children with comorbid sensory processing disorder (SPD) and inattention symptoms [18]. These children further improved on clinical ADHD inattention symptoms as measured by the Vanderbilt parent-report survey [19]. Finally, a recently published, large randomized controlled trial extended this work to children with ADHD and a demonstrated behavioral attention impairment [20]. This trial showed that AKL-T01 significantly improved objective behavioral measures of attention compared to an expectancy-matched digital control intervention and also improved clinical ADHD-related symptoms and impairments, although not significantly greater than those in the control group [20]. Importantly, however, intervention-related changes in brain function were not examined in this trial.

Thus, while there is growing evidence that AKL-T01 can improve objective behavioral metrics of attentional control in youth with attention difficulties, the underlying neural changes associated with these improvements have not yet been examined in children with ADHD who do not have other comorbidities. AKL-T01 may be particularly effective at improving neural markers of attentional control (MFT) in this population, given the overlap of this circuit with brain regions affected in those with ADHD. Specifically, MFT is believed to be generated by the midcingulate cortex, a brain network hub with strong interconnections to various cortical and sub-cortical regions [14]. MFT signals can therefore entrain other brain networks over large spatial distances when behavioral control is required [14], such as the fronto-parietal, attention, and other control networks affected in ADHD [21].

To address this gap, we sought to examine intervention-related changes in a neural marker of attentional control (MFT) in a cohort of children meeting diagnostic criteria for ADHD. Specifically, we examined AKL-T01 improvements in a broad, heterogenous population of children with ADHD and did not selectively include those with objective attention difficulties at baseline, as other AKL-T01 studies [20]. Our primary analyses focused on MFT activity, where we hypothesized that children with ADHD would show MFT enhancement following 4 weeks of at-home treatment with AKL-T01. We also conducted exploratory analyses examining intervention-related improvements on objective behavioral metrics of attentional control and clinical ADHD symptoms.

## Materials and methods

### Participants and design

In this study, we enrolled 28 children with a confirmed ADHD diagnosis (based on DSM-5 criteria), between March 2019 to February 2020. Characterization of clinical ADHD symptoms in children often relies upon parent report questionnaires for ease of administration [22–24]. As such, ADHD inclusion criteria for this study was based on the Vanderbilt Parent-Report measure as a screening tool [19]. Following the Vanderbilt criteria for inattention symptoms in ADHD (inattention subscale), parents had to report a symptom frequency of ‘often’ or ‘very often’ (2 or 3 on a scale from 0-‘never’ to 3-‘very often’) on at least six of nine items relating to inattention. In addition, we confirmed this ADHD diagnosis with the Mini-International Neuropsychiatric Interview for Children and Adolescents (MINI Kid, Section N [25]), administered by a trained clinician.

Exclusion criteria were brain malformation or injury, movement disorders, psychiatric conditions (e.g., bipolar, psychotic, and autism spectrum disorders), and hearing impairment. In addition, children had to be off antipsychotic or ADHD medications (stimulants, alpha-adrenergic medications, or atomoxetine) for the duration of the study and a washout period of at least 30 days prior to initiation. Further, children had to score ≥ 70 on the Perceptual Reasoning Index (PRI) on the Wechsler Intelligence Scale for Children–Fifth Edition and had to be able to comply with all testing and requirements. Participants who were eligible were then assessed with a battery of behavioral and neural outcome measures, both prior to and no later than 7 days after the 4-week AKL-T01 intervention.

Participants were recruited from online parent groups, the Sensory Neurodevelopment and Autism Program research registry, and Cortica Healthcare, Marin Center. The study is registered with ClinicalTrials.gov (NCT03844269) as a prospective trial and has approval through WIRB Copernicus Group (IRB Tracking Number: 20190399, Study Number: 1254952). Participants’ primary caregivers provided written consent on behalf of their child and children provided informed assent.

### Primary outcome metric: Neural assessment of attentional control

#### EEG data collection and preprocessing

Participants performed a perceptual discrimination task that assessed selective attention abilities with concurrent electroencephalography (EEG) recording, as in our previous work [18]. The perceptual discrimination task was a go/no-go task where participants were instructed to respond to specific stimuli (green circles) presented on a computer monitor while ignoring all other color and shape combinations. The task was comprised of 3 blocks of 36 target stimuli and 36 non-target stimuli, with each stimulus appearing on the screen for 400 ms and an inter-trial interval of 2000–3000 ms (with 500 ms jitter). A fixation cross was present on the screen at all times and also provided performance feedback–it turned green for 50 ms when participants responded correctly (i.e., responding to green circles within the time-window or ignoring irrelevant stimuli) and it turned red for 50 ms for when participants responded incorrectly.

Electroencephalography (EEG) activity was recorded with a BioSemi ActiveTwo 64-channel EEG acquisition system in conjunction with BioSemi ActiView software (Cortech-Solutions). Signals were amplified and digitized at 1024 Hz with a 16-bit resolution. Anti-aliasing filters were applied and the data were band-pass filtered between 0.01–100 Hz during acquisition. Data was preprocessed using Analyzer software (Brain Vision, LLC), with blinks and eye-movement artifacts removed through an independent component analysis, as were epochs with excessive peak-to-peak deflections (± 100 μV).

#### ERSP analysis

The cleaned EEG data were exported into EEGLAB for event-related spectral perturbation (ERSP) analysis. As in our previous work [17, 18], epochs of -1000 to +1000 ms time-locked to stimulus onset were created for ERSP total power analysis (evoked power + induced power), with theta band activity analyzed by resolving 4–100 Hz activity using a complex Morlet wavelet and referenced to a -900 to -700 pre-stimulus baseline (relative power (dB)). To examine changes in MFT power, we assessed activity at the same frontal composite electrodes of interest (EOI) as in our previous work [17, 18], from the mean of AFz, Fz, FPz, AF3 and AF4 electrodes. However, visual inspection revealed consistent voltage artifacts across participants at the FPz electrode, leading to our decision to remove it from all analyses. To balance the localization of the EOI, electrodes F1 and F2 were added to the composite mean, as visual inspection of these electrodes collapsed across sessions showed comparable time courses of activity as the original four (i.e., Fz, AFz, AF3 and AF4).

All ERSP data was segmented into 40 ms time bins from -400 ms to +880 ms, where time 0 represents the onset of the target stimulus. Here, we focused on post-stimulus ERSP changes in peak MFT, as in our previous work [18]. In addition to peak MFT, other work has pointed to the relevance of early and late EEG-based components in populations with cognitive control and attentional difficulties, such as ADHD [26–29]. To examine potential timing differences across the 0–880 ms time course, we created 120 ms composite bins at 3 timepoints during the trials: (1) the first three windows (‘early’: 0–120 ms), (2) the three windows around the MFT peak (‘peak’: 160–280 ms, as the peak time bin was found to be 200–240 ms, see **Results**), and (3) the last three windows (‘late’: 760–880 ms). We created these longer, 120 ms composite bins (as opposed to looking at singular 40 ms time points) to better account for multiple comparison concerns and potential deviations at each of these *a priori* composite time windows.

### Exploratory outcome metrics: Objective behavioral measures of attention

To assess objective behavioral improvements from AKL-T01, we used two computerized tasks to probe components of attentional control abilities.

#### Perceptual discrimination task

In addition to intervention-related neural changes during the perceptual discrimination task, we assessed concurrent changes in task performance. We quantified changes in response time (RT) and response time variability (RTV), in line with our previous work in children [18].

#### Sustained attention task

To further index improvements in attentional control, we assessed changes on a visual continuous performance task (CPT) similar to the Test of Variables of Attention (TOVA [30]) used in previous *NeuroRacer* and AKL-T01 studies [17, 18, 20]. This task was a 22-minute, fixed interval, CPT administered on a laptop computer. Participants were instructed to respond to a visual stimulus (white square) appearing at the top edge of the computer screen (target stimuli) and to withhold responses to a white square appearing at the bottom edge of the screen (non-target stimuli). The task has two conditions: (1) a sustained attention condition, where the target stimuli are infrequent (appear in 22% of trials) and (2) an inhibitory/impulsivity condition, where the target stimuli are frequent (appears in 77% of trials). Here, we focused on the sustained attention condition and assessed intervention-related changes in RT and RTV, in line with our work in children with SPD [18]. Further, we also assessed changes in a metric related to RTV that quantifies attentional lapses by examining the distribution of long RTs–ex-Gaussian tau [31, 32]. Work in children and adolescents with attention difficulties has shown that they have poorer performance on this related, yet distinct, RT metric [33, 34]. Unlike other studies examining the efficacy of AKL-T01 in children with ADHD [20], we did not use baseline performance on this task as part of the study inclusion criteria (i.e., we did not selectively include participants who exhibited a performance impairment on this task).

### Exploratory outcome metrics: Parent-reported ADHD symptoms

#### Vanderbilt ADHD diagnostic parent scale

The Vanderbilt is a standardized clinical parent-report survey used to assess ADHD symptoms. Parent reports of clinically significant inattention symptoms (inattention subscale) were used for inclusion criteria in this study to demonstrate ADHD symptomatology (see **Participants and Design**). In addition, we assessed intervention-related changes of clinical ADHD inattention symptoms by re-administering this scale to the same parent/caregiver after the intervention and analyzing changes in symptom severity on the inattention subscale.

### AKL-T01 intervention

The AKL-T01 intervention is a game-based digital therapeutic developed by Akili Interactive, which implements proprietary algorithms designed to train attentional control by enhancing interference management (multi-tasking) at an adaptive and personalized degree of difficulty. In brief, the AKL-T01 intervention is a self-guided at-home intervention that involves both a perceptual discrimination targeting task, in which users respond to the instructed stimulus targets and ignore the stimulus distractors (similar to a go/no-go task), and a sensory motor navigation task, in which users continuously adjust their location to interact with or avoid positional targets. Performance in each task is assessed during single and multitask conditions.

The AKL-T01 intervention was preloaded and administered on iPad Mini 2 tablets (Apple, USA). Children were instructed to complete 5 ‘missions’ at least 5 days per week for 4 weeks for a total of approximately 25 minutes of gameplay per day (at least 100 missions). Adherence was monitored remotely by study coordinators using an electronic dashboard created by Akili. If a participant had more than two incomplete days of the intervention, a reminder phone call was made to the parents, while also providing support and feedback to the parents and children with the intervention. Additionally, AKL-T01 generates automatic reminders. After a participant completed 20 sessions of the AKL-T01 intervention, a follow-up research appointment was scheduled with the parents. All data, except for the AKL-T01 intervention, was collected at the Cortica Neurodevelopment Centers, Marin Campus.

### Statistical analysis

Changes in our primary outcome metric—neural measures of attentional control (MFT EEG power)—were assessed with a repeated-measures ANOVA with within-subjects factors of session (pre and post), composite 120 ms time-windows (early, peak, and late), and electrode (Fz, AFz, AF3, AF4, F1, and F2). Given that we aimed to replicate our previous analytical approaches and had no *a priori* hypotheses about unique effects of electrode, all MFT analyses presented are collapsed along the factor of electrode (i.e., we averaged electrode values after all other processing steps). In addition to this approach with a within-subjects factor of time-window, we also interrogated each 120 ms window separately, given that MFT activity at these windows may reflect different ongoing cognitive processes throughout the duration of the task trials. To do so, we conducted 3 separate ANOVAs for each time window, with a within-subjects factor of session (pre and post).

Improvements on exploratory metrics of attentional control tasks and survey-based measures were assessed with paired t-tests comparing pre- and post-intervention performance. When examining intervention-related changes, we calculated gain scores for each metric, where higher values indicate improvements in performance or neural activity. To examine relationships between neural improvements and behavioral and clinical improvements, we performed Pearson correlations between neural and cognitive and clinical gains scores. For statistically significant correlations, we also report the strength of the correlation as ‘very strong’ (r = 0.9–1.0), ‘strong’ (r = 0.70–0.89), ‘moderate’ (r = 0.40–0.69), ‘weak’ (r = 0.10–0.39), or ‘negligible’ (r < 0.10).

All statistical analyses were conducted using SPSS 24.0 (SPSS Inc.). We report significant effects at a threshold of p ≤ 0.05 and also report marginal ‘trends’ at p ≤ 0.10. Further, effect sizes for intervention-related changes are reported as Cohen’s d, where 0.2 = small effect, 0.5 = medium effect, and 0.8 = large effect [35]. We did not correct for multiple comparisons for each test conducted, as our primary goal was to examine neural markers of attentional control and our exploratory analyses aimed to replicate findings using similar metrics in patients with inattention and SPD [18].

## Results

### Participant demographics

**Fig 1:**
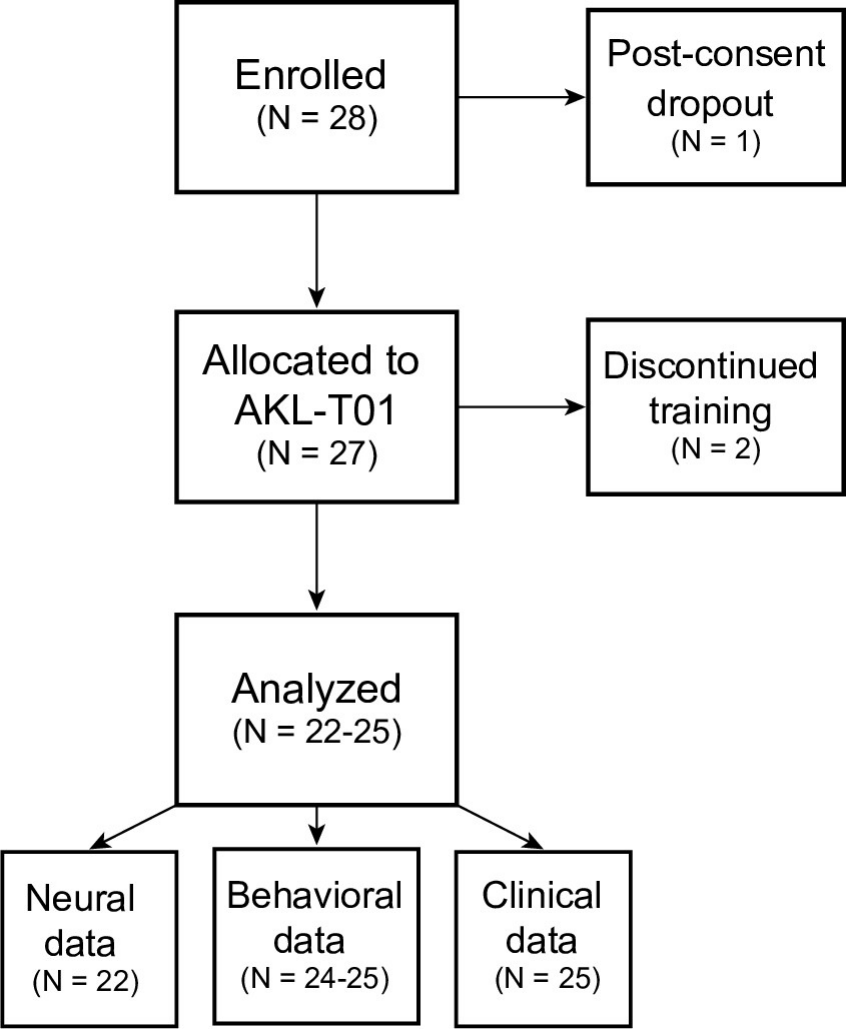
CONSORT flow diagram.

Twenty-eight participants meeting our inclusion and exclusion criteria were enrolled in the study. Twenty-five of these participants successfully completed the assessments and intervention (age mean ± SD: 10.44 ± 1.23 years old, age range: 8–12; number of males: 20, 80% of total sample). Of the 3 participants that did not complete the study, one participant withdrew during the pre-intervention baseline assessments and two participants did not complete the intervention (Fig 1). The 25 participants that completed the study had normal performance on the Wechsler Intelligence Scale for Children (WISC-V, see S1 Table in S1 File) and showed excellent adherence with the AKL-T01 intervention (percentage of ‘missions’ completed: 100 ± 20%, range = 61–136%). Note that participants could have opted to play 7 days a week (5 days were recommended) and additionally extend the gameplay until the scheduled outcome assessment post-intervention (when they returned their devices). Due to technical difficulties during data collection, outcome measurement data was not available for all 25 participants. Further, EEG data from 2 participants was excluded due to excessive noise (rejection of more than 30% of target trials due to voltage fluctuations greater than ± 100 μV deflections within an epoch [36, 37]) at the pre- or post-intervention assessment (available data: EEG N = 22, perceptual discrimination task N = 25, sustained attention task N = 24, Vanderbilt parent-report survey N = 25).

### Primary outcome metric: Intervention-related changes in neural markers of attentional control

**Fig 2:**
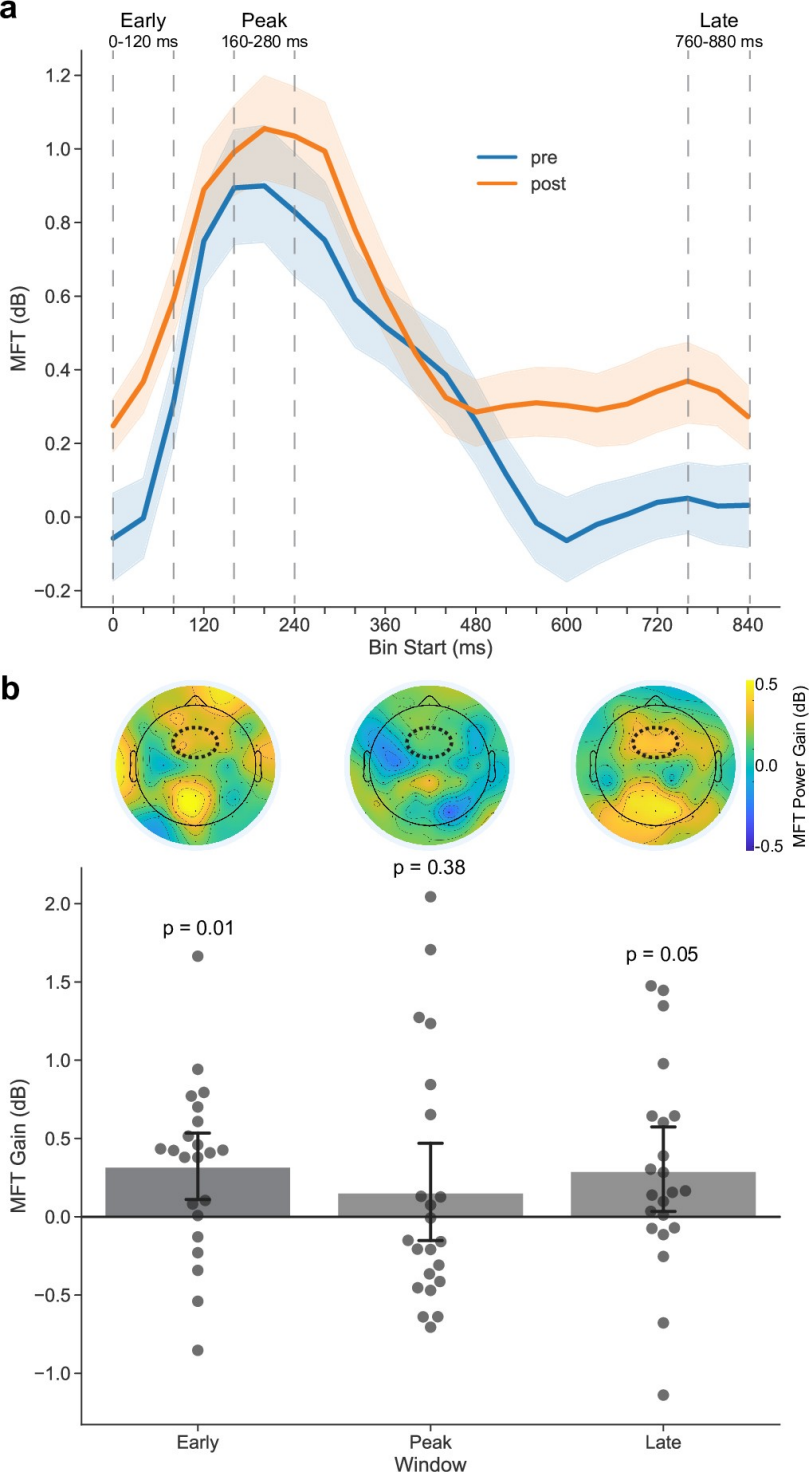
Changes in primary outcome metrics: Neural markers of attention. **a** Time course of midline frontal theta (MFT) pre- and post-intervention at each 40 ms time bin during the perceptual discrimination task, where 0 ms represents stimulus onset. Solid lines represent the mean and shaded areas represent 95% bootstrapped confidence intervals (1000 bootstrap iterations). **b** Intervention-related improvements in MFT at early, peak, and late 120 ms composite windows, also illustrated through topographic heat maps with the 6 electrodes of interest highlighted with a dotted bounding box. Data are presented as mean ± 95% bootstrapped confidence intervals (1000 bootstrap iterations), and circles represent individual participants. P-values represent repeated-measures ANOVAs comparing pre- and post-intervention MFT at each composite window.

To interrogate effects of AKL-T01 on overall attention-related neural activity during the perceptual discrimination task, we conducted a repeated-measures ANOVA on the MFT EEG data, with within-subjects factors of session (pre and post) and 120 ms composite time-window (early, peak, and late post-stimulus onset). We observed a main effect of session, (F(1,21) = 4.76, p = 0.04, d = 0.44), suggesting that there was a general increase in MFT following the intervention (Fig 2A). We did not observe an interaction between session and composite time-window (F(2,42) = 0.78, p = 0.46, d = 0.11). However, given our *a priori* hypotheses that the intervention may affect the cognitive processes occurring at each window differently, we next examined each 120 ms composite window separately. To do so, we conducted 3 repeated-measures ANOVAs with a within-subjects factor of session, separately for each time window. We found increases in the early (0–120 ms: F(1,21) = 7.53, p = 0.01, d = 0.62) and late (760–880 ms: F(1,21) = 4.51, p = 0.046, d = 0.52) time windows. There was a numerical increase in MFT activity at the peak time period of 160–280 ms following stimulus onset, however in contrast to our previous work [17, 18], this change did not reach significance (F(1,21) = 0.82, p = 0.38, d = 0.19) (Fig 2B).

### Exploratory outcome metrics: Intervention-related changes in objective behavioral measures of attention

#### Perceptual discrimination task

**Fig 3:**
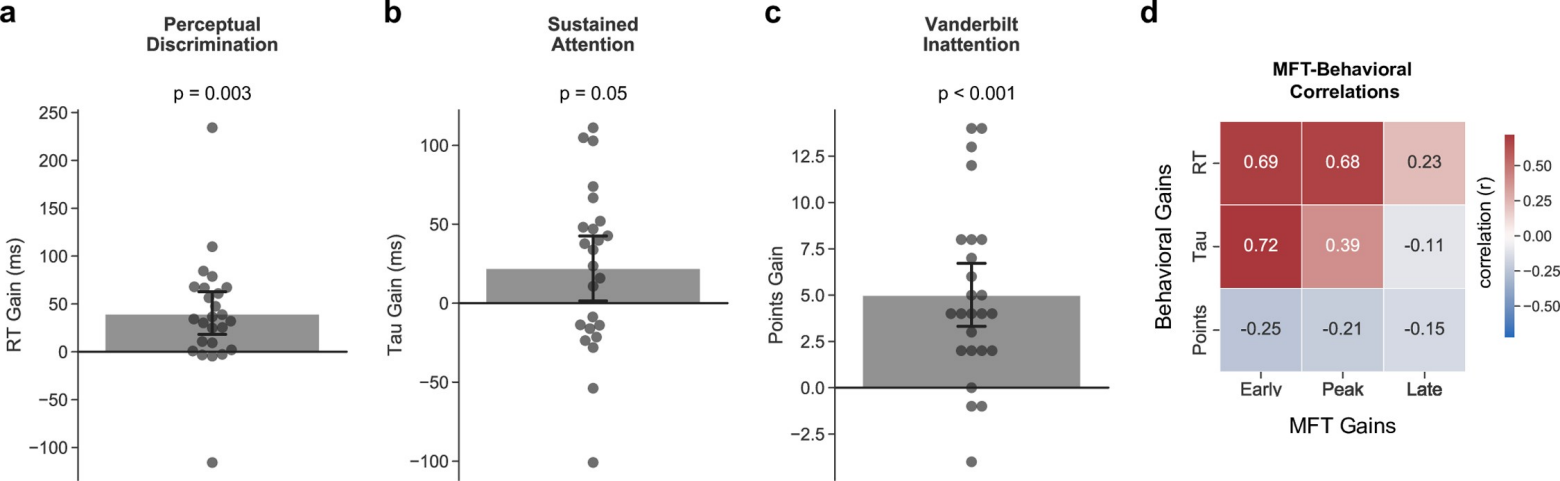
Changes in exploratory outcome metrics: Behavioral and parent-report measures of attention. Intervention-related changes in objective behavioral and parent-report measures of attention, specifically **a** response time (RT) on the perceptual discrimination task, **b** tau on the sustained attention task, and **c** points on the Vanderbilt inattention subscale (parent-report). Data are presented as mean ± 95% bootstrapped confidence intervals (1000 bootstrap iterations), and circles represent individual participants. **d** Pearson correlations between midline frontal theta (MFT) improvements at each composite window and the behavioral and parent-report improvements.

For the perceptual discrimination task, we observed an improvement on response time (RT) (mean ± SE gain = 39.67 ± 11.86 ms; t(1,24) = 3.35, p = 0.003, d = 0.62), suggesting that participants performed faster on this measure following AKL-T01 (Fig 3A). However, we did not observe an improvement on response time variability (RTV; t(1,24) = -0.76, p = 0.45, d = 0.12) (see S2 Table in S1 File for full descriptive statistics for each metric).

#### Sustained attention task

For the sustained attention task, we did not observe improvements on RT or RTV (t(1,23) = 0.35, p = 0.73, d = 0.09 and t(1,23) = 0.14, p = 0.89, d = 0.03, respectively). However, we did observe an improvement on tau (mean ± SE gain = 22.08 ± 10.58 ms; t(1,23) = 2.09, p = 0.048, d = 0.46), suggesting that participants had fewer attentional lapses following AKL-T01 (Fig 3B) (see S2 Table in S1 File for full descriptive statistics for each metric).

#### Consideration of potential confounds

We confirmed that intervention-related changes on these measures were not driven by changes in basic motoric speed (see Supplementary Information). Briefly, basic response time (BRT) changes were not related to any other training-related neural or behavioral changes, but had a marginal relationship with clinical inattention symptoms. Further, controlling for BRT changes did not affect results for clinical inattention improvements after AKL-T01 (see below).

### Exploratory outcome metrics: Intervention-related changes in parent reports of clinical inattention symptoms

To test whether AKL-T01 led to changes in ADHD inattention symptoms, we examined changes in our clinical outcome measure–the inattention subscale of the Vanderbilt parent report. After the AKL-T01 intervention, parents observed significantly fewer ADHD inattention symptoms (mean ± SE gain = 5.00 ± 0.94 points; t(1,24) = 5.32, p < 0.001, d = 1.25), suggesting that participants had fewer clinical inattention symptoms following the intervention (Fig 3C). Furthermore, 44% (11 participants out of 25) of individuals who previously met inclusion criteria (≥ 6 out of 9 Vanderbilt inattention symptoms in the ‘often’ or ‘very often’ range) no longer met this cutoff after AKL-T01.

### Relationships between neural and behavioral changes

To examine associations between training-related neural changes and the observed behavioral and clinical improvements, we performed correlations between neural gain scores at the three composite time-windows and improvements on the behavioral and survey-based assessments (Fig 3D). Notably, changes in RT on the perceptual discrimination task were related to early and peak post-stimulus MFT gains (r(21) = 0.69, p < 0.001 and r(21) = 0.68, p = 0.001, respectively; both ‘moderately’ sized correlations), but not to late MFT gains (r(21) = 0.23, p = 0.31). Further, we also found that early and peak post-stimulus MFT gains were related to changes in tau on the sustained attention task (r(20) = 0.72, p < 0.001 and r(20) = 0.39, p = 0.08, respectively; ‘strong’ and ‘weak’ correlations, respectively), but not to late MFT gains (r(20) = -0.11, p = 0.65). We did not, however, find any relationships between MFT and Vanderbilt inattention improvements.

## Discussion

The present findings demonstrate that a targeted, digital therapeutic (AKL-T01) can have wide-ranging positive effects on several metrics of attention in a heterogenous population of children with ADHD. Specifically, our primary analyses show that AKL-T01 enhances midline frontal theta (MFT), a well-established EEG-based measure of attentional control. Further, our exploratory analyses demonstrate that AKL-T01 improves performance on computerized tasks of attention as well as clinical ADHD symptoms from parent reports. Here, we discuss the possible mechanisms of these intervention-related enhancements.

### Intervention-related changes in neural markers of attentional control

First, we demonstrate that AKL-T01 increases MFT broadly following post-stimulus presentation in children with ADHD, suggesting that these patients are better able to engage control functions and monitor ongoing cognitive processes to support attention-centric performance. Other neuromodulatory approaches, such as neurofeedback training, have also pointed to similar plasticity of MFT, by showing that targeted neurofeedback can broadly enhance MFT activity during training as well as during ‘baseline’ periods [38]. Critically, this shift in MFT concurrently improves behavioral attentional control abilities that require proactive control [39]. In addition, non-invasive neurostimulation applied in the theta frequency benefits complex cognitive functions, such as interference processing [40]. The findings of the present study expand on this work and suggest that a digital therapeutic targeting specific brain networks is an effective form of neuromodulation that can broadly enhance MFT activity and attention-related behavior.

In addition to general MFT improvements during task performance, we also observed that these neural changes were most pronounced in the early and late stages of the perceptual discrimination task trials. Examining each time window separately, MFT changes were most evident immediately following stimulus presentation (‘early’) and in preparation for the upcoming task trial (‘late’), although it should be noted that we did not observe a formal statistical interaction between session and time window for these analyses. Early processing (approximately 100 ms post-stimulus presentation) is thought to reflect initial orienting of attentional resources [41–43], while late processing (approximately 650–800 ms post-stimulus presentation) may reflect post-response processes, such as suppressing the previous stimulus and updating in preparation for the upcoming trial [26]. With respect to the present study, children with ADHD have shown impaired neural oscillations for both early and late stimulus processing [26]. The neural findings reported here demonstrate that AKL-T01 improves MFT signals supporting attentional control in children with ADHD, particularly (1) early processes related to attentional orienting and (2) late processes related to future event preparation. It is important to note that, unlike previous studies in older adults and children with SPD and inattention [17, 18], we did not observe significant changes in peak MFT. However, given that the children in this study did exhibit numerical peak MFT gains that also were related to positive changes in task performance, the lack of a significant intervention effect may be a function of statistical power or the heterogenous ADHD population in the present study. Future research with larger sample sizes may help confirm the presence of peak MFT improvements in this population.

### Intervention-related changes in objective behavioral and clinical metrics of attention

In addition to the intervention-related neural improvements in MFT activity, we also replicate previous work demonstrating that AKL-T01 can improve both behavioral and clinical measures of attention in children with inattention difficulties [18, 20]. We further demonstrate that improved performance on these computer-based tasks was not simply due to general improvements in motoric speed, indicating that AKL-T01 improved indices of more complex attentional behaviors.

Critically, improvements on these computerized measures were also related to improvements on our EEG-based metric of attention (MFT activity), particularly for the ‘early’ and ‘peak’ time windows. These results suggest that there are benefits to including neuroimaging markers as an adjunct to assessing behavioral and clinical outcome measures, and further point to a potential underlying neural mechanism of the observed changes in behavior. Interestingly, these correlational results also point to individual differences in intervention-related improvements–children that exhibit the greatest neural changes are those that improve the most behaviorally. These findings signal the potential importance for developing personalized interventions that enhance improvements at the individual patient level.

Finally, we also found that AKL-T01 improved clinical measures of ADHD–the Vanderbilt parent report used for inclusion criteria of ADHD inattention symptoms. This finding is consistent with previous work using AKL-T01 interventions in children with attention difficulties and indicate that, in this study, children with ADHD had fewer parent-reported symptoms of inattention after the intervention. Notably, almost half (44%) of the children no longer met inclusion criteria for Vanderbilt inattention symptoms after the intervention. This is in line with our previous report that 33% of children with SPD and inattention showed this level of improvement following training [18]. These results provide compelling evidence that AKL-T01 has benefits that generalize beyond computerized tasks, and also reduces inattention behaviors observed by parents in real-world settings.

## Limitations and conclusions

While this study represents an effort to expand our understanding of intervention-related neural enhancements in a population of children meeting clinical ADHD criteria, future work is warranted to address limitations of this current study. First, comparisons to a mechanistic or placebo control group would help elucidate the extent of such intervention-related improvements, as in previous work [20]. Second, it is unclear how long such neural effects would persist and how they would relate to the aforementioned behavioral measures of attention. We recently demonstrated that clinical improvements of inattention symptoms on the Vanderbilt parent report persist 3 years after the AKL-T01 intervention in children with SPD and inattention [44], but did not obtain neurophysiologic or objective behavioral data to elucidate these effects. Furthermore, we recently showed that intervention-related increases in MFT activity persist 6 years after the *NeuroRacer* intervention in older adults [45], suggesting that such interventions have potential for both immediate and long-lasting positive outcomes on behavior and brain function. Finally, it is important to note that the reported neural-behavioral gain correlations were not corrected for multiple comparisons, given our primary focus on identifying intervention-related neural changes. In addition, the participants in this study represented a heterogenous population of children with ADHD with varying attention difficulties prior to the AKL-T01 intervention, unlike other studies that excluded children whose objective attention functioning was not significantly impaired [20]. Future work with larger samples can replicate the neural-behavioral gain correlations and also more directly examine the role of baseline abilities in predicting intervention-related improvements.

The present findings contribute to the recent burst of empirical work aiming to demonstrate the efficacy of various digital health technologies. Here, we provide supporting evidence for the efficacy of one such digital therapeutic in remediating attentional control difficulties in children with ADHD. We show that AKL-T01 improves behavioral and clinical metrics of attention, replicating previous work [18, 20]. Importantly, we demonstrate that underlying changes in a neural signature of attentional control (MFT) are related to such behavioral benefits, thus pointing to neural mechanisms of these changes. Future work that further examines which children benefit most from this intervention will be critical to developing customized interventions to maximize such beneficial effects.

## Supporting information

S1 Checklist CONSORT checklist. (DOC)

S1 Protocol Study protocol developed by investigators and study sponsor (Akili Interactive, Inc). (PDF)

S1 File Supplementary information. (DOCX)
