# Supplementary material for: Enhancing neural markers of attention in children with ADHD using a digital therapeutic
Source: PLoS One. 2021 Dec 31;16(12):e0261981. doi: 10.1371/journal.pone.0261981 (PMC8719702; doi:10.1371/journal.pone.0261981)
Supplement: S1 Protocol — (PDF) [file pone.0261981.s002.pdf]

A study to assess Midline Frontal Theta (MFT) power as measured by stimulus-locked electroencephalography (EEG) before and after AKL-T01 treatment for improving attention in pediatric participants ages 8-12 years old with Attention Deficit Hyperactivity Disorder (ADHD)

Sponsor Protocol ID: 001S-C

Sponsor: Akili Interactive Labs, Inc

Principal Investigator: Elysa J. Marco, MD

Version Number: 1.0

February 8, 2019

PROTOCOL APPROVAL PAGE

Study Title: A study to assess Midline Frontal Theta (MFT) power as measured by stimulus-locked electroencephalography (EEG) before and after AKL-T01 treatment for improving attention in pediatric participants ages 8-12 years old with Attention Deficit Hyperactivity Disorder (ADHD)

Version: 1.0

Date of Issue: February 8, 2019

Study Sponsor: Akili Interactive, Inc.
125 Broad St., 4th FL
Boston, MA 02110

We, the undersigned, have read and approve this protocol and agree on its content.

DocuSigned by:
Elysa Marco
96E0FFB39CCD46C... 2/11/2019

Elysa J. Marco, MD
Principal Investigator

DocuSigned by:
A Jina
7913908FE1BE4B9... 2/11/2019

Anil Jina, MD
Sponsor – Medical Affairs

DocuSigned by:
Scott Kellogg
AD704F59F0DF470... 2/8/2019

Scott Kellogg
Sponsor – Clinical Operations

DocuSigned by:
Joaquin Anguera
1C0EF0EE14CF49B... 2/8/2019

Joaquin A. Anguera, PhD
Assistant Professor – UCSF

Summary of Changes from Previous Version:

Affected Section(s)	Summary of Revisions Made	Rationale

Table of Contents

STATEMENT OF COMPLIANCE 1
1 List of Abbreviations 2
1 PROTOCOL SUMMARY 3
1.1 Synopsis 3
1.2 Schema 4
1.3 Schedule of Activities (SoA) 5
2 INTRODUCTION 5
2.1 Study Rationale 5
2.2 Background 6
2.3 Risk/Benefit Assessment 6
2.3.1 Known Potential Risks 6
2.3.2 Known Potential Benefits 7
2.3.3 Assessment of Potential Risks and Benefits 7
3 OBJECTIVES AND ENDPOINTS 7
4 STUDY DESIGN 7
4.1 Overall Design 7
4.2 Justification for Dose 8
5 STUDY POPULATION 8
5.1 Inclusion Criteria 8
5.2 Exclusion Criteria 9
6 STUDY INTERVENTION 9
6.1 Study Intervention 9
6.1.1 Study Intervention Description 9
6.1.2 Study Intervention Compliance 10
6.2 Concomitant Medication and Treatment 10
7 STUDY INTERVENTION DISCONTINUATION AND PARTICIPANT DISCONTINUATION/WITHDRAWAL 10
7.1 Discontinuation of Study Intervention 10
7.1.1 Participant Discontinuation or Withdrawal 10
7.1.2 Lost to Follow-Up 11
8 STUDY ASSESSMENTS AND PROCEDURES 11
8.1 Eligibility and Efficacy Assessments 11
8.2 Safety and Other Assessments 12
8.3 Adverse Events and Serious Adverse Events 12
8.3.1 Definition of Adverse device effects (AdE) 12
8.3.2 Definition of unanticipated Adverse device effects (uade) 12
8.3.3 previously known adverse device effects 13
8.3.4 Classification of an Adverse Event 13
8.3.5 Time Period and Frequency for Event Assessment and Follow-Up 14
8.3.6 Adverse event Reporting 14
8.4 Unanticipated Problems 14
8.4.1 Definition of Unanticipated Problems (UP) 14
8.4.2 Unanticipated Problem Reporting 15
9 STATISTICAL CONSIDERATIONS 16
9.1 Statistical Hypotheses 16
9.2 Populations for Analyses 16

9.3 Statistical Analyses 16
10 SUPPORTING DOCUMENTATION AND OPERATIONAL CONSIDERATIONS 16
10.1 Confidentiality and Privacy 17
10.2 Data Handling and Record Keeping 17
10.2.1 Data Collection and Management Responsibilities 17
10.2.2 Study Records Retention 17
10.3 Protocol Deviations 17
11 REFERENCES 18

STATEMENT OF COMPLIANCE

The trial will be carried out in accordance with International Conference on Harmonization Good Clinical Practice (ICH GCP) and the following:

- United States (US) Code of Federal Regulations (CFR) applicable to clinical studies (45 CFR Part 46, 21 CFR Part 50, 21 CFR Part 56, 21 CFR Part 312, and/or 21 CFR Part 812)

The protocol, informed consent form(s), recruitment materials, and all participant materials will be submitted to the Institutional Review Board (IRB) for review and approval. Approval of both the protocol and the consent form must be obtained before any participant is enrolled. Any amendment to the protocol will require review and approval by the IRB before the changes are implemented to the study. In addition, all changes to the consent form will be IRB-approved; a determination will be made regarding whether a new consent needs to be obtained from participants who provided consent, using a previously approved consent form.

1 LIST OF ABBREVIATIONS

	List in alphabetical order
ADHD	Attention-Deficit Hyperactivity Disorder
AE	Adverse Event
API	Attention Performance Index
CFR	Code of Federal Regulations
CPT	Continuous Performance Test
CRF	Case Report Form
eCRF	Electronic Case Report Form
EEG	Electroencephalography
FDA	Food and Drug Administration
FDAAA	Food and Drug Administration Amendments Act of 2007
GCP	Good Clinical Practice
GMP	Good Manufacturing Practices
HIPAA	Health Insurance Portability and Accountability Act
ICF	Informed Consent Form
ICH	International Conference on Harmonization
ICMJE	International Committee of Medical Journal Editors
IDE	Investigational Device Exemption
IND	Investigational New Drug Application
IRB	Institutional Review Board
MedDRA	Medical Dictionary for Regulatory Activities
NCT	National Clinical Trial
NT	Neurotypical
PI	Principle Investigator
SAE	Serious Adverse Event
SAP	Statistical Analysis Plan
SOA	Schedule of Activities
TOVA	Test of Variables of Attention

1 PROTOCOL SUMMARY

1.1 SYNOPSIS

Title: A study to assess Midline Frontal Theta (MFT) power as measured by stimulus-locked electroencephalography (EEG) before and after AKL-T01 treatment for improving attention in pediatric participants ages 8-12 years old with Attention Deficit Hyperactivity Disorder (ADHD)

Hypothesis: ADHD children receiving 4 weeks of AKL-T01 treatment will show enhancement in MFT activity as measure by stimulus-locked EEG.

Objectives:

Primary Objective:

- Demonstrate enhancement of MFT power measured by stimulus-locked EEG (Perceptual Discrimination Task (PDT)=Stimulus) in children with ADHD following 4 weeks of at-home AKL-T01 treatment.

Secondary Objective:

- Demonstrate normalized MFT power in children with ADHD following 4 weeks of at-home AKL-T01 treatment.

Exploratory Objectives:

- Demonstrate improvement in sustained attention by direct assessment in children with ADHD following 4 weeks of at-home AKL-T01 treatment.
- Demonstrate improvement in ADHD symptoms by parent report in children with ADHD following 4 weeks of at-home AKL-T01 treatment.

Endpoints:

Primary Endpoint:

- PDT-locked EEG relative to pre-training

Secondary Endpoint:

- PDT-locked EEG relative to a normative control cohort

Exploratory Endpoints:

- Project EVO Assessment
- Test of Variables of Attention (TOVA)
- Vanderbilt Assessment

Study Population: 30 children ages 8 to 12 with ADHD from the United States

Description of Study Intervention: AKL-T01 is a digital therapeutic software for the treatment of attention and inhibitory control deficits in patients with ADHD. AKL-T01 is an investigational medical device known as Software as a Medical Device (SaMD).

Study Duration: From beginning of enrollment to last subject last visit, 12 months

Participant Duration: 29 Days

1.2 SCHEMA

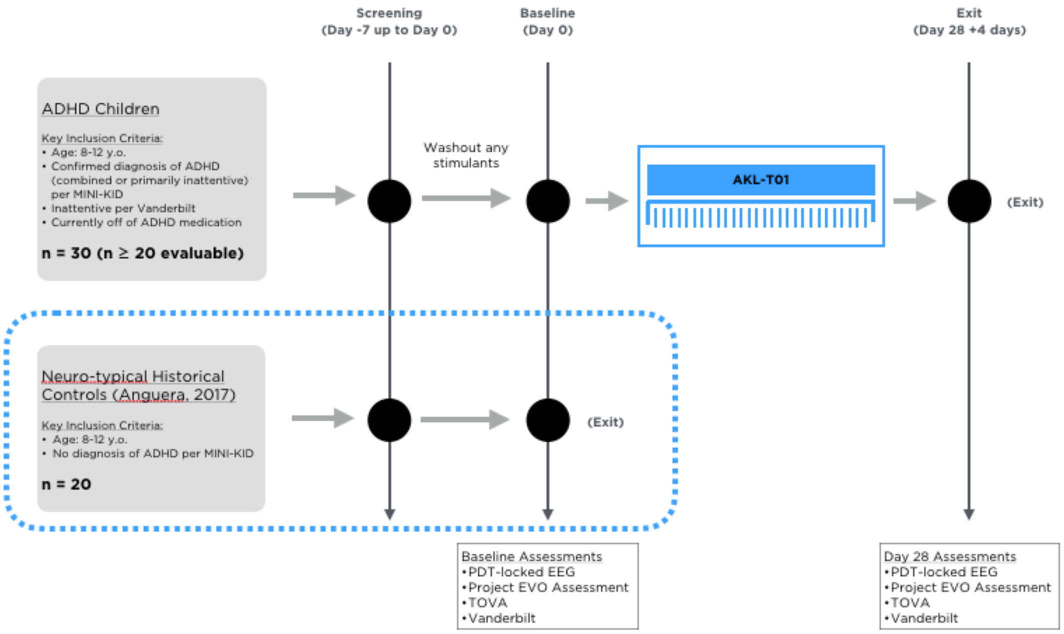
Screening
(Day -7 up to Day 0)
Baseline
(Day 0)
Exit
(Day 28 +4 days)
ADHD Children
Key Inclusion Criteria:
• Age: 8-12 y.o.
• Confirmed diagnosis of ADHD (combined or primarily inattentive) per MINI-KID
• Inattentive per Vanderbilt
• Currently off of ADHD medication
n = 30 (n ≥ 20 evaluable)
Washout any stimulants
AKL-T01
(Exit)
Neuro-typical Historical Controls (Anguera, 2017)
Key Inclusion Criteria:
• Age: 8-12 y.o.
• No diagnosis of ADHD per MINI-KID
n = 20
(Exit)
Baseline Assessments
•PDT-locked EEG
•Project EVO Assessment
•TOVA
•Vanderbilt
Day 28 Assessments
•PDT-locked EEG
•Project EVO Assessment
•TOVA
•Vanderbilt

1.3 SCHEDULE OF ACTIVITIES (SOA)

Procedures	Screening* Day -7 to -3 (Participants washing out stimulants only)	Enrollment / Baseline* Day 0	Treatment Days 1 to 27	Follow Up Day 28 +3 days
Parent Informed consent		X		
Child Assent		X		
Mini-International Neuropsychiatric Interview for Children and Adolescents (MINI-KID)		X		
Demographics	X			
Medical history	X			
Wechsler Intelligence Scale for Children V (WISC V)		X		
Ishihara Colorblindness Test		X		
Vanderbilt ADHD Diagnostic Parent Rating Scale (Initial and Follow-Up)		X		X
Social Communication Questionnaire		X		
Short Sensory Profile		X		
Social Competence Scale		X		X
Video Game Play Questionnaire		X		
Behavior Assessment for Children, Second Edition (BASC-2)		X		
Beery Visual Motor Integration (VMI)		X		
Adaptive Cognitive Evaluation (ACE)†		X		X
Neuroracer†-locked EEG		X		X
Resting State EEG		X		X
AKL-M01 (AKL-T01 Monitor) †		X		X
Test of Variables pf Attention (TOVA)†		X		X
Basic Response Time (BRT)		X		X
Administer AKL-T01		X		
AKL-T01 Compliance Monitoring			X	
Adverse Event (AE) Assessment				X
Complete Case Report Forms (CRFs)	X	X	X	X

* Screening and Baseline visits may be a combined visit for participants not washing out stimulant medication.
† Interventions are computerized

2 INTRODUCTION

2.1 BACKGROUND

Akili Interactive's videogame-like digital treatment, AKL-T01™ (EVO™-Multi), is based on the work of UCSF Professor Adam Gazzaley, MD, who has identified a way to measure and improve the ability to process interference events (essentially, interruptions and distractions). Interference susceptibility is recognized as a limiting factor across global executive function (including attention and memory) and is known to be fragile in multiple diseases. Dr. Gazzaley's work also demonstrated that changes in EEG signals occurred at neurological loci associated with cognitive control. The basic scientific mechanisms underlying AKL-T01 have been tested in controlled (N=203) studies of healthy participants across a wide age range (8 to 70 years of age) to validate the ability to detect differences between age groups and to enhance interference processing in older adults. AKL-T01 was tested in a, follow-on, controlled (N=80) study of ADHD children and age-matched neurotypical controls to validate its ability to detect differences between the groups and to provide insight into AKL-T01 as a therapeutic product. Findings provide preliminary support that this digital treatment may improve attention, working memory, and inhibition in children with ADHD. The pivotal study STARS-ADHD; a multi-center, randomized, double-blind, active-controlled study of AKL-T01 in 348 pediatric subjects ages 8-12 with ADHD demonstrated a statistically significant improvement (p=006) between AKL-T01 and an active control on the primary endpoint (TOVA API change from Baseline to Post-Treatment) (STARS-ADHD, in progress).

Most relevant to this protocol is a published feasibility study of pediatric Sensory Processing Dysfunction (SPD at the University of California, San Francisco (UCSF), which requested that 57 children ages 8 to 12 (24 Neurotypical, 20 SPD with inattention, and 13 SPD without inattention) to engage with AKL-T01 treatment at home for 30 minutes per day, 5 days per week for 4 weeks. Participants were assessed using EEG, TOVA and the Vanderbilt Inattention scale (parent-reported) at Baseline/Day 0 and at Exit/Day 28. All children showed statistically significant improvement in attention as measured by TOVA. Children with SPD and with inattention also showed statistically significant enhancement of Mid-Frontal Cortex Theta power as measured by EEG while neurotypical and SPD children without inattention did not.

2.2 STUDY RATIONALE

Previous work has been done to establish the effect of AKL-T01 on MFT activity in children with SPD and inattention according to the Vanderbilt ADHD Diagnostic Parent Rating Scale, however no work has been done to study the effect of MFT activity in children with a confirmed diagnosis of ADHD.

2.3 RISK/BENEFIT ASSESSMENT

2.3.1 KNOWN POTENTIAL RISKS

The risks to the participants in this study are very small. The study involves completing some computerized tests, game-based digital therapies, and answering some questions. It is possible that the participant could become frustrated or fatigued by some of the tasks.

The participant may also experience dizziness, headaches, nausea, decreased frustration tolerance, or emotional reaction as a result of playing the study device. If the participant becomes frustrated or

fatigued, they may stop at any time. There are no other risks to taking part in this study of which we are aware.

2.3.2 KNOWN POTENTIAL BENEFITS

Based on preliminary data obtained in several studies, including a feasibility study of AKL-T01 in pediatric SPD subjects, the potential benefits of the AKL-T01 game-based treatment for this population may include improvements in midline frontal theta activity and/or parental report of inattention. Given the central role of attention in the pathophysiology of ADHD, this benefit is substantial.

2.3.3 ASSESSMENT OF POTENTIAL RISKS AND BENEFITS

To date, there have been no serious adverse events reported across several studies with the device. The risk/benefit ratio for use is favorable.

3 OBJECTIVES AND ENDPOINTS

OBJECTIVES	ENDPOINTS
Primary	
Demonstrate enhancement of MFT power measured by stimulus-locked EEG (Perceptual Discrimination Task (PDT)=Stimulus) in children with ADHD following 4 weeks of at-home AKL-T01 treatment.	Change in midline frontal theta power as measured by PDT-locked EEG.
Secondary	
Demonstrate normalized MFT power in children with ADHD following 4 weeks of at-home AKL-T01 treatment.	MFT power of ADHD cohort at Day 28 compared to that of neurotypical cohort at Day 0 (historical controls).
Exploratory	
Demonstrate improvement in attention in children with ADHD following 4 weeks of at-home AKL-T01 treatment.	• Reaction time and reaction time variability as measured by TOVA.<br>• Reaction time and reaction time variability as measured by perceptual discrimination task.<br>• Reaction time and reaction time variability as measured by ALK-M01 Assessment.

4 STUDY DESIGN

4.1 OVERALL DESIGN

This study is a single arm, open-label, pilot study to assess midline frontal theta (MFT) power as measured by stimulus-locked electroencephalogram (EEG) before and after treatment with AKL-T01 for improving attention in pediatric participants ages 8-12 years old with attention deficit hyperactivity disorder (ADHD).

All participants enrolled in this study will not be taking ADHD medications, including stimulants, for the duration of the study. Participants who are taking ADHD medications prior to Day 0 must have been stable off of medications for at least 30 days per parent report, or washout of medications at a Screening visit 3 – 7 days prior to Day 0.

At Baseline / Day 0, all eligible participants will complete both resting-state and PDT-locked EEG, as well as the TOVA, Vanderbilt, and AKL-M01 assessments.

All participants will then play AKL-T01 for approximately 25 minutes per day, 5 days per week, for 4 weeks at home.

At Follow-up / Day 28, participants will repeat both resting-state and PDT-locked EEG, as well as the TOVA, Vanderbilt, and AKL-M01 assessments.

All study visits for each participant should be completed during the same time of day (either AM or PM), to maintain consistent data collection and comparison of rater assessment results.

4.2 JUSTIFICATION FOR DOSE

The therapeutic play regimen for the current study (typically 25 minutes per day; 5 days per week; 4 weeks) was established based on previous work with AKL-T01 digital treatment. Specifically, a recent pivotal trial of AKL-T01 demonstrated that this regimen resulted in significant improvements in TOVA API scores in a sample of children with attention and inhibitory control deficits (STARS-ADHD).

5 STUDY POPULATION

5.1 INCLUSION CRITERIA

In order to be eligible to participate in this study, an individual must meet all of the following criteria:

1. Age 8 to 12 at the time of parental informed consent
2. Male or female
3. Confirmed ADHD diagnosis at screening/baseline visit based on DSM-V criteria and established via the MINI-KID administered by a trained clinician either in person or via teleconference
4. WISC V FSIQ ≥ 70
5. Vanderbilt ADHD Diagnostic Parent Rating Scale: Must score a 2 or 3 on at least 6 items 1-9 AND must score a 4 on at least 2, or 5 on at least 1, of items 48-54 (performance questions).
6. Consistently off stimulant medication for ≥ 1 week. OR currently on stimulant medication and agree to stop taking the medication for a 1 week prior to the baseline visit and off through duration of training and post-training assessment (note: participants will only be allowed to washout of stimulant medication if in the opinion of the investigator they are currently inadequately managed on their medication and it is appropriate to stop taking their medication for the duration of the trial)
7. Consistently off Psychotropic drug for ≥ 1 month
8. Consistently off non-stimulant medication for ADHD (e.g. atomoxetine, clonidine, guanfacine) for ≥ 1 month

9. Able to follow written and verbal instructions (English) as assessed by the PI and/or study coordinator
10. Functioning at an age-appropriate level intellectually
11. Able to comply with all testing and requirements

5.2 EXCLUSION CRITERIA

An individual who meets any of the following criteria will be excluded from participation in this study:

1. Current controlled (requiring a restricted medication) or uncontrolled, comorbid psychiatric diagnosis, based on the Neurodevelopment Intake Form, BASC, and subsequent clinical interviewing, with significant symptoms including but not limited to post-traumatic stress disorder, psychosis, bipolar illness, severe obsessive compulsive disorder, severe depressive or anxiety disorder, conduct disorder, or other symptomatic manifestations that in the opinion of the Investigator may confound study data/assessments. (Participants with clinical history of learning disorders will be allowed to participate as long as the disorder does not impact their ability to participate based on PI judgement).
2. Autism Spectrum Disorder concern as indicated from the Social Communication Questionnaire ≥ 15.
3. Current treatment with stimulant treatment for ADHD and unwilling or inappropriate (per investigator opinion) to washout.
4. Initiation or completion of behavioral therapy within the last 4 weeks. The participant should inform the Investigator if they intend to change their behavioral therapy during the 4 weeks of the study. Participants who have been in behavior therapy consistently for more than 4 weeks may participate if their routine is unchanged throughout the study.
5. Participant is currently considered at risk for attempting suicide by the Investigator, has previously made a suicide attempt, or has a prior history of, or is currently demonstrating active suicidal ideation or self-injurious behavior, as measured by MINI-KID Suicidality Module C.
6. Motor condition (e.g. physical deformity of the hands/arms) that prevents game playing as reported by the parent or observed by the Investigator.
7. Recent history or suspicion (within the past 6 months) of substance abuse or dependence.
8. History of seizures (excluding febrile seizures).
9. Participation in a clinical trial within 90 days prior to screening.
10. Color blindness as detected by Ishihara Color Blindness Test.
11. Regular use of psychoactive drugs that in the opinion of the Investigator may confound study data/assessments.
12. Any other medical condition that in the opinion of the Investigator may confound study data/assessments.
13. Previously received AKL-T01 (Project-EVO™) treatment in a previous clinical trial.

6 STUDY INTERVENTION

6.1 STUDY INTERVENTION

6.1.1 STUDY INTERVENTION DESCRIPTION

AKL-T01 digital treatment is a state-of-the-art mobile video game-like platform, which deploys modern videogame graphics, engaging reward loops, and real-time adaptive mechanics to dynamically personalize difficulty based on the user's ability. AKL-T01 multitasking treatment employs perceptual discrimination attention/memory task as well as a continuous motor "driving" task. Performance on these tasks are assessed in isolation and when performed together to calculate a performance index for each individual user. A personalized multitask treatment regimen is automatically configured and delivered to the user and is optimized adaptively to increase multitask performance. As players proceed through the treatment periodic recalibration occurs to maintain an optimal difficulty level.

6.1.2 STUDY INTERVENTION COMPLIANCE

AKL-T01 automatically captures gameplay compliance and uploads these data directly to a central server when connected to WiFi. The server will automatically push daily compliance emails to the research coordinator. Based on these emails study staff can then reach out to parents and caregivers of participants to trouble shoot technical problems and/or encourage more play.

Additionally, AKL-T01 contains built-in features that remind the participant to play each day.

6.2 CONCOMITANT MEDICATION AND TREATMENT

With the exception of common over the counter (OTC) (e.g. ibuprofen, acetaminophen, non-sedating antihistamines to treat seasonal allergies) and prescription medications (e.g. antibiotics) for minor transient ailments, regular use of concomitant medications are not permitted during the study.

Concomitant medications and treatment are as follow:

1. Psychotropic medication
2. Stimulant medication
3. Non-stimulant medication for treatment of ADHD (e.g. atomoxetine, clonidine, guanfacine)

Transient use of some non-sedating antihistamines is allowed.

Study Investigators can approve short-term use of other medications that are not anticipated to confound study assessments.

7 STUDY INTERVENTION DISCONTINUATION AND PARTICIPANT DISCONTINUATION/WITHDRAWAL

7.1 DISCONTINUATION OF STUDY INTERVENTION

AKL-T01 study intervention may be discontinued by the participant or investigator at any time during the study.

7.1.1 PARTICIPANT DISCONTINUATION OR WITHDRAWAL

Participants are free to withdraw from participation in the study at any time upon request without prejudice.

An investigator may discontinue or withdraw a participant from the study for the following reasons:

- Disease progression which requires discontinuation of the study intervention
- If the participant meets an exclusion criterion (either newly developed or not previously recognized) that precludes further study participation
- Participant unable to receive AKL-T01 for 5 days/weeks as prescribed per protocol
- If any clinical adverse event (AE) or other medical condition or situation occurs such that continued participation in the study would not be in the best interest of the participant

The investigator or designee will notify the sponsor or their designee immediately when a subject has been discontinued or withdrawn from study treatment because of an adverse experience. Any adverse experiences that are present at the time of discontinuation/withdrawal should be reported and followed up in accordance with the safety requirements outlined in Section 8.3 (Adverse Events).

7.1.2 LOST TO FOLLOW-UP

A participant will be considered lost to follow-up if he or she fails to return for Day 28 Exit visit and is unable to be contacted by the study site staff.

The following actions must be taken if a participant fails to return to the clinic for a required study visit:

- The site will attempt to contact the participant and reschedule the missed Follow-up / Day 28 visit.
- Before a participant is deemed lost to follow-up, the investigator or designee will make every effort to regain contact with the participant.
- Should the participant continue to be unreachable, he or she will be considered to have withdrawn from the study with a primary reason of lost to follow-up.

8 STUDY ASSESSMENTS AND PROCEDURES

8.1 ELIGIBILITY AND EFFICACY ASSESSMENTS

The following assessments will be administered to each eligible participant

Assessment	Purpose	Time to Administer
Eligibility		
MINI-KID	Confirm diagnosis of ADHD and identify any excluding comorbid diagnoses	20 minutes
WISC V	Determine IQ	60 minutes
Ishihara Colorblindness Test	Determine colorblindness	5 minutes
Efficacy		

Assessment	Purpose	Time to Administer
Vanderbilt ADHD Diagnostic Parent Rating Scale	Assess severity of ADHD symptoms and effect on behavior.	20 minutes
Resting State EEG	Assess MFT activity in a resting state	6 min
PDT-locked EEG	Assess MFT activity during a Perceptual Discrimination Task	1 hour
Test of Variables of Attention (TOVA)	Assess objective attention	22 minutes
AKL-M01 (EVO Monitor)	Assess MFT activity during a AKL-M01 session	20 minutes

8.2 SAFETY AND OTHER ASSESSMENTS

Safety assessments will include the evaluation of adverse device effects (ADEs).

8.3 ADVERSE EVENTS AND SERIOUS ADVERSE EVENTS

8.3.1 DEFINITION OF ADVERSE DEVICE EFFECTS (ADE)

An ADE is an adverse event related to the use of an investigational medical device. This includes any adverse event resulting from insufficiencies or inadequacies in the instructions for use, the deployment, the implantation, the installation, the operation, or any malfunction of the investigational medical device. This also includes any event that is a result of a user error or intentional misuse.

8.3.2 DEFINITION OF UNANTICIPATED ADVERSE DEVICE EFFECTS (UADE)

Per 21 CFR 812.3, an Unanticipated Adverse Device Effect is any serious adverse effect on health or safety or any life-threatening problem or death caused by, or associated with, a device, if that effect, problem, or death was not previously identified in nature, severity, or degree of incidence in the investigational plan or application (including a supplementary plan or application), or any other unanticipated serious problem associated with a device that relates to the rights, safety, or welfare of participants.

Unanticipated Adverse Device Effects will include events meeting BOTH A and B as stated below:

A. Events meeting ALL of the following criteria:
   1) Not included in the list of Anticipated Events (refer to protocol Section 8.3.3)
   2) Possibly, probably, or definitely related to the investigational device (per the sponsor)

B. Serious (meets any of the following criteria):
   1) Is life-threatening illness or injury
   2) Results in permanent impairment of a body function or a body structure
   3) Necessitates medical or surgical intervention to prevent permanent* impairment of a body function or a body structure. (***Permanent*** is defined as irreversible impairment or damage to a body structure or function, excluding trivial impairment or damage)
   4) Leads to fetal distress, fetal death or a congenital abnormality or birth defect
   5) Leads to death.

8.3.3 PREVIOUSLY KNOWN ADVERSE DEVICE EFFECTS

The expectedness of an ADE shall be determined according to the Instructions for Use and the expected ADEs listed below. Any ADE that is not identified in nature, severity, or specificity in the current investigational product reference documents is considered unanticipated.

Adverse Device Effects expected for this device are as follows:

- Dizziness
- Nausea
- Headache
- Decreased frustration tolerance
- Emotional reaction

8.3.4 CLASSIFICATION OF AN ADVERSE EVENT

8.3.4.1 SEVERITY OF EVENT

The following guidelines will be used to describe severity of an ADE:

- **Mild** – Events require minimal or no treatment and do not interfere with the participant's daily activities.
- **Moderate** – Events result in a low level of inconvenience or concern with the therapeutic measures. Moderate events may cause some interference with functioning.
- **Severe** – Events interrupt a participant's usual daily activity and may require systemic drug therapy or other treatment. Severe events are usually potentially life-threatening or incapacitating. Of note, the term "severe" does not necessarily equate to "serious".

8.3.4.2 RELATIONSHIP TO STUDY TREATMENT

All ADEs must have their relationship to study treatment assessed by the clinician who examines and evaluates the participant based on temporal relationship and his/her clinical judgment. In a clinical trial, the investigational device must always be suspect. The degree of certainty of causal relationship of an adverse device effect of either study treatment will be rated as follows:

- **Possible:** An event that might be due to the use of the study device. An alternative explanation, e.g., concomitant drug(s), concomitant disease(s), is inconclusive. The relationship in time is reasonable; therefore, the causal relationship cannot be excluded.
- **Probable:** An event that might be due to the use of the study device. An alternative explanation is less likely - e.g., concomitant drug(s), concomitant disease(s). The relationship in time is suggestive.
- **Definitely:** An event that is due to the use of the study device. The event cannot be reasonably explained by an alternative explanation - e.g., concomitant drug(s), concomitant disease(s).

8.3.4.3 EXPECTEDNESS

Akili Interactive Labs, Inc will be responsible for determining whether an adverse event (AE) is expected or unexpected. An ADE will be considered unexpected if the nature, severity, or frequency of the event is not consistent with the risk information previously described for the study treatment.

8.3.5 TIME PERIOD AND FREQUENCY FOR EVENT ASSESSMENT AND FOLLOW-UP

The occurrence of an ADE may come to the attention of study personnel during study visits and interviews of a study participant presenting for medical care, or upon review by a study monitor.

Information to be collected includes event description, time of onset, clinician's assessment of severity, relationship to study product (assessed only by those with the training and authority to make a diagnosis), and time of resolution/stabilization of the event. All ADEs occurring while on study will be documented appropriately regardless of relationship. All ADEs will be followed to adequate resolution.

Any medical condition that is present at the time that the participant is screened will be considered as baseline and not reported as an ADE. However, if the study participant's condition deteriorates at any time during the study, it will be recorded as an ADE.

Changes in the severity of an ADE will be documented to allow an assessment of the duration of the event at each level of severity to be performed. ADEs characterized as intermittent require documentation of onset and duration of each episode.

The Site Principal Investigator will record all reportable events with start dates occurring from the time of enrollment through the final study visit. At each study visit, the investigator will inquire about the occurrence of ADEs since the last visit. Events will be followed for outcome information until resolution or stabilization.

8.3.6 ADVERSE EVENT REPORTING

It is understood that complete information about an event may not be known at the time the initial report is submitted. The investigator must assess the relationship of the event to the investigational device (including rationale for assessment) and should make every attempt to obtain as much information as possible concerning the event. Additional information pertaining to an event should be reported in the case report form as it becomes available.

The investigator must report all adverse device effects (anticipated or unanticipated) occurring from the time of enrollment through the final study visit within 24 hours of knowledge of the event.

The investigator must report when important follow-up information (final diagnosis, outcome, results of specific investigations, etc.) becomes available after submission of the initial ADE form and information. Follow-up information should be submitted according to the same process used for reporting the initial event as described above (i.e., within 24 hours of knowledge, via <specify method>). All adverse device effects will be followed through resolution.

8.4 UNANTICIPATED PROBLEMS

8.4.1 DEFINITION OF UNANTICIPATED PROBLEMS (UP)

The Office for Human Research Protections (OHRP) considers unanticipated problems involving risks to participants or others to include, in general, any incident, experience, or outcome that meets **all** of the following criteria:

- Unexpected in terms of nature, severity, or frequency given (a) the research procedures that are described in the protocol-related documents, such as the Institutional Review Board (IRB)-approved research protocol and informed consent document; and (b) the characteristics of the participant population being studied;
- Related or possibly related to participation in the research ("possibly related" means there is a reasonable possibility that the incident, experience, or outcome may have been caused by the procedures involved in the research); and
- Suggests that the research places participants or others at a greater risk of harm (including physical, psychological, economic, or social harm) than was previously known or recognized.

In addition, UPs include unanticipated adverse device effect, any serious adverse effect on health or safety, or any life-threatening problem or death caused by, or associated with, a device, if that effect, problem, or death was not previously identified in nature, severity, or degree of incidence in the investigational plan or application (including a supplementary plan or application), or any other unanticipated serious problem associated with a device that relates to the rights, safety, or welfare of subjects (21 CFR 812.3(s)).

- Examples of potential UPs include but are not limited to:
    1. Unanticipated misuse of device hardware
    2. Unanticipated misuse of device software
    3. Unanticipated hardware issues
    4. Unanticipated software issues

8.4.2 UNANTICIPATED PROBLEM REPORTING

The investigator will report unanticipated problems (UPs) to the reviewing Institutional Review Board (IRB) and to the Data Coordinating Center (DCC)/lead principal investigator (PI). The UP report will include the following information:

- Protocol identifying information: protocol title and number, PI's name, and the IRB project number;
- A detailed description of the event, incident, experience, or outcome;
- An explanation of the basis for determining that the event, incident, experience, or outcome represents an UP;
- A description of any changes to the protocol or other corrective actions that have been taken or are proposed in response to the UP.

An investigator shall submit to the sponsor and to the reviewing Institutional Review Board (IRB) a report of any unanticipated adverse device effect occurring during an investigation as soon as possible, but in no event later than 10 working days after the investigator first learns of the effect (21 CFR 812.150(a)(1)). A sponsor who conducts an evaluation of an unanticipated adverse device effect under 812.46(b) shall report the results of such evaluation to the Food and Drug Administration (FDA) and to all reviewing IRBs and participating investigators within 10 working days after the sponsor first receives

notice of the effect. Thereafter the sponsor shall submit such additional reports concerning the effect as FDA requests (21 CFR 812.150(b)(1)).

9 STATISTICAL CONSIDERATIONS

9.1 STATISTICAL HYPOTHESES

OBJECTIVE	HYPOTHESIS
Primary	Children with ADHD, following 4 weeks of digital treatment with AKL-T01, will demonstrate enhancement of MFT power as measured by stimulus-locked EEG.
Secondary	Children with ADHD, following 4 weeks of digital treatment with AKL-T01, will demonstrate normalized MFT power as compared with a neurotypical population.

9.2 POPULATIONS FOR ANALYSES

There will be two populations for analysis in this study.

The AKL-T01 Intervention Population will complete 28 days of AKL-T01 digital treatment. This population will enroll pediatric ADHD participants according to the inclusion criteria outlined in section 5.1 of this protocol. Only participants completing at least 50% of the prescribed treatment (i.e. 50 sessions) will be included in the analysis.

The neurotypical control group will also be analyzed as historical controls. This population was enrolled in *A pilot study to determine the feasibility of enhancing cognitive abilities in children with sensory processing dysfunction* (Anguera, 2017).

9.3 STATISTICAL ANALYSES

All statistical analyses will be conducted using SPSS 22.0 (SPSS Inc.). Group effects for both behavioral and neural measures will be assessed with separate ANOVAs and ANCOVAs with planned follow-up contrasts (Fisher's LSD). The ANCOVA approach is considered to be a more suitable approach when post-test performance that is not conditional / predictable based on pre-test performance is the primary outcome of interest following treatment, as opposed to characterizing gains achieved from pre-training performance (for example, group X session interaction(s). However, both are appropriate statistical approaches that have been used to assess cognitive training outcomes. Thus, our approach will be to present the p-values for each approach. Planned follow-up contrasts will be used to directly assess changes within each group. To minimize influential data points, we will remove values +/- 2 standard deviations for all group analyses. For correlations tested, we will use a more stringent outlier removal procedure (Cook's D> 1) given the smaller cohort size and possible inflated change scores.

10 SUPPORTING DOCUMENTATION AND OPERATIONAL CONSIDERATIONS

10.1 CONFIDENTIALITY AND PRIVACY

Participant confidentiality and privacy is strictly held in trust by the participating investigators, their staff, and the sponsor(s) and their interventions. The study protocol, documentation, data, and all other information generated will be held in strict confidence. No information concerning the study or the data will be released to any unauthorized third party without prior written approval of the sponsor.

10.2 DATA HANDLING AND RECORD KEEPING

10.2.1 DATA COLLECTION AND MANAGEMENT RESPONSIBILITIES

Data collection is the responsibility of the clinical trial staff at the site under the supervision of the principal investigator. The investigator is responsible for ensuring the accuracy, completeness, legibility, and timeliness of the data reported.

All source documents should be completed in a neat, legible manner to ensure accurate interpretation of data. Data recorded in the electronic case report form (eCRF) derived from source documents should be consistent with the data recorded on the source documents.

10.2.2 STUDY RECORDS RETENTION

Study documents should be retained for a minimum of 2 years after the last approval of a marketing application in an International Conference on Harmonization (ICH) region and until there are no pending or contemplated marketing applications in an ICH region or until at least 2 years have elapsed since the formal discontinuation of clinical development of the study intervention. These documents should be retained for a longer period, however, if required by local regulations. No records will be destroyed without the written consent of the sponsor. It is the responsibility of the sponsor to inform the investigator when these documents no longer need to be retained.

10.3 PROTOCOL DEVIATIONS

A protocol deviation is any noncompliance with the clinical trial protocol, ICH GCP, or Manual of Procedures (MOP) requirements. The noncompliance may be either on the part of the participant, the investigator, or the study site staff. As a result of deviations, corrective actions are to be developed by the site and implemented promptly.

11 REFERENCES

Anguera, J. A., Boccanfuso, J., Rintoul, J. L., Al-Hashimi, O., Faraji, F., Janowich, J., ... & Gazzaley, A. (2013). Video game training enhances cognitive control in older adults. *Nature*, *501*(7465), 97.

Anguera, J. A., Gunning, F. M., & Areán, P. A. (2016). Improving late life depression and cognitive control through the use of therapeutic video game technology: A proof-of-concept randomized trial. *Depression and anxiety*, *34*(6), 508-517.

Anguera, J. A., Brandes-Aitken, A. N., Antovich, A. D., Rolle, C. E., Desai, S. S., & Marco, E. J. (2017). A pilot study to determine the feasibility of enhancing cognitive abilities in children with sensory processing dysfunction. *PloS one*, *12*(4), e0172616.
