# Supplementary material for: Enhancing neural markers of attention in children with ADHD using a digital therapeutic
Source: PLoS One. 2021 Dec 31;16(12):e0261981. doi: 10.1371/journal.pone.0261981 (PMC8719702; doi:10.1371/journal.pone.0261981)
Supplement: S1 File — (DOCX) [file pone.0261981.s003.docx]

**Enhancing neural markers of attention in children with ADHD using a digital therapeutic**

Courtney L. Gallen, Ph.D.^1, 2¶^, Joaquin A. Anguera, Ph.D.*^1, 2, 3¶^ , Molly R. Gerdes, B.S.^4^, Alexander J. Simon, B.S.^1, 2^, Elena Cañadas, Ph.D.^5^, and Elysa J. Marco, M.D.^4,6^

**Supplementary Information**

**Supplementary Table S1.** Descriptive statistics for the WISC-V

|  | Mean | SD |
| --- | --- | --- |
| Verbal Comprehension Index (VCI) | 109.92 | 14.15 |
| Processing Speed Index (PSI) | 90.88 | 9.53 |
| Working Memory Index (WMI) | 96.00 | 16.67 |
| Fluid Reasoning Index (FRI) | 105.38 | 13.50 |
| Visual Spatial Index (VSI) | 104.92 | 14.65 |
| Full scale IQ (FSIQ) | 103.38 | 14.22 |

**Supplementary Table S2.** Descriptive statistics for behavioral and parent-report data

|  | Pre-Intervention | | Post-Intervention | |
| --- | --- | --- | --- | --- |
|  | Mean | SD | Mean | SD |
| Perceptual Discrimination RT (ms) | 531.58 | 75.66 | 491.91 | 53.28 |
| Perceptual Discrimination RTV (ms) | 59.60 | 14.66 | 61.38 | 14.70 |
| Sustained Attention RT (ms) | 449.30 | 65.13 | 443.02 | 82.08 |
| Sustained Attention RTV (ms) | 155.18 | 36.21 | 153.87 | 50.16 |
| Sustained Attention Tau (ms) | 151.95 | 38.53 | 129.87 | 57.56 |
| Vanderbilt Inattention Score | 22.16 | 3.69 | 17.16 | 4.32 |

**Consideration of potential confounds**

## As in our previous work[18], we also administered a Basic Response Time (BRT) task as a measure of simple motoric response time to ensure that any differences observed between groups were not due to differences in basic motoric quickness. In this task, participants responded to a target stimulus (40 trials) by tapping a button with their dominant hand.

We observed a significant improvement on BRT RT (mean ± SE gain = 90.43 ± 38.10 ms, t(1,23) = 2.37, p = 0.03), suggesting that participants performed faster on this measure following the intervention. However, we conducted correlations between BRT RT gains and the behavioral and neural metrics and found that BRT RT gains were only marginally related to Vanderbilt gains (r(23) = 0.39, p = 0.06), but were not related to any of the other behavioral or EEG measures that exhibited intervention-related changes (p = 0.14-0.72). Additionally, the improvements on Vanderbilt remained significant after controlling for participant BRT RT gains (assessed with a repeated-measures ANOVA with a within-subjects factor of time, controlling for BRT RT gain: F(1,24) = 22.00, p = 0.001) Importantly, this demonstrates that the changes in motoric quickness were not related to, or driving, the intervention-related improvements in behavior and underlying neural activity.
